# Mapping of DDX11 genetic interactions defines sister chromatid cohesion as the major dependency

**DOI:** 10.1093/g3journal/jkae052

**Published:** 2024-03-13

**Authors:** Leanne Amitzi, Ecaterina Cozma, Amy Hin Yan Tong, Katherine Chan, Catherine Ross, Nigel O’Neil, Jason Moffat, Peter Stirling, Philip Hieter

**Affiliations:** Michael Smith Laboratories, University of British Columbia, 2185 East Mall, Vancouver, British Columbia, V6T 1Z4, Canada; Terry Fox Laboratory, BC Cancer Research Institute, 675 West 10th Avenue, Vancouver, British Columbia, V5Z 1L3, Canada; Donnelly Centre, University of Toronto, Toronto, Ontario, M5S 3E1, Canada; Donnelly Centre, University of Toronto, Toronto, Ontario, M5S 3E1, Canada; Donnelly Centre, University of Toronto, Toronto, Ontario, M5S 3E1, Canada; Michael Smith Laboratories, University of British Columbia, 2185 East Mall, Vancouver, British Columbia, V6T 1Z4, Canada; Donnelly Centre, University of Toronto, Toronto, Ontario, M5S 3E1, Canada; Department of Molecular Genetics, University of Toronto, Toronto, Ontario, M5S1A8, Canada; Institute of Biomedical Engineering, University of Toronto, Toronto, Ontario, M5S3E1, Canada; Terry Fox Laboratory, BC Cancer Research Institute, 675 West 10th Avenue, Vancouver, British Columbia, V5Z 1L3, Canada; Michael Smith Laboratories, University of British Columbia, 2185 East Mall, Vancouver, British Columbia, V6T 1Z4, Canada

**Keywords:** DDX11, helicase, synthetic lethality, cohesin, CRISPR

## Abstract

DDX11/Chl1R is a conserved DNA helicase with roles in genome maintenance, DNA replication, and chromatid cohesion. Loss of DDX11 in humans leads to the rare cohesinopathy Warsaw breakage syndrome. DDX11 has also been implicated in human cancer where it has been proposed to have an oncogenic role and possibly to constitute a therapeutic target. Given the multiple roles of DDX11 in genome stability and its potential as an anticancer target, we set out to define a complete genetic interaction profile of DDX11 loss in human cell lines. Screening the human genome with clustered regularly interspaced short palindromic repeats (CRISPR) guide RNA drop out screens in DDX11-wildtype (WT) or DDX11-deficient cells revealed a strong enrichment of genes with functions related to sister chromatid cohesion. We confirm synthetic lethal relationships between *DDX11* and the tumor suppressor cohesin subunit *STAG2*, which is frequently mutated in several cancer types and the kinase HASPIN. This screen highlights the importance of cohesion in cells lacking DDX11 and suggests DDX11 may be a therapeutic target for tumors with mutations in *STAG2*.

## Introduction

Synthetic lethality (SL) occurs when a sublethal genetic defect allows cell viability but leads to death if combined with a second sublethal genetic defect. Given the potential specificity of such interactions, SL is touted as a solution for genotype targeted precision anticancer therapeutics (Hartwell *et al*. 1997; O’Neil *et al*. 2017). The approval of poly-ADP ribose polymerase (PARP) inhibitors for the treatment of *BRCA1/2*-mutated advanced ovarian cancers and more recently for *BRCA1/2*-mutated breast cancers illustrates the potential of SL-based therapeutics (Bryant *et al*. 2005; Farmer *et al*. 2005; Kim *et al*. 2015). Cohesin is mutated across diverse cancer types, including glioblastoma, bladder cancer, acute myeloid leukemia, and Ewing's sarcoma (Solomon *et al*. 2014; Romero-Pérez *et al*. 2019). In total, this amounts to well over 100,000 Americans per year affected by a cohesin-mutated cancer, raising the possibility that cohesin–SL interactions could be impactful therapeutically. Previously, we screened for SL with mutant forms of cohesin subunits in yeast and identified a hub of SL targets, all associated with the stability of the replication fork (McLellan *et al*. 2009, 2012). One of the potentially druggable targets is the helicase Chl1 (the yeast ortholog of human DDX11 helicase).

DDX11 is a superfamily 2, an ATP-dependent DEAH/DEAD-box containing helicase belonging to the XPD-like helicase family, which contains 4 members (FANCJ, XPD, RTEL1, and DDX11), all having a conserved Fe–S-binding domain (Bharti *et al*. 2014). These proteins play important roles in genome stability and are implicated in rare genetic syndromes and cancer development (Wu *et al*. 2009; Suhasini and Brosh 2013). In vitro DDX11 unwinds DNA/DNA and DNA/RNA duplexes, as well as G-quadruplex (G4) structures with a preferred 5′ to 3′ directionality (Hirota and Lahti 2000; Farina *et al*. 2008; Wu *et al*. 2012; Bharti *et al*. 2013). DDX11 also interacts with Ctf18-RFC, PCNA, and FEN1 and stimulates FEN1 endonuclease activity on a flap DNA structure, a model intermediate substrate that forms during lagging strand synthesis (Farina *et al*. 2008).

Mutations in the yeast ortholog *chl1* result in elevated levels of chromosome loss or missegregation (Gerring *et al*. 1990; Holloway 2000). Chl1 plays a critical role during establishment of proper sister chromatid cohesion during S-phase and in DNA repair (Petronczki *et al*. 2004; Skibbens 2004; Ogiwara *et al*. 2007; Chung *et al*. 2011; Stoepker *et al*. 2015; Abe *et al*. 2018). In mammalian cells, depletion of DDX11 by shRNA/siRNA results in aneuploidy, abnormal sister chromatid cohesion, and a prometaphase delay leading to mitotic failure (Parish *et al*. 2006). DDX11 is required for proper chromosome cohesion at both centromeres and along the chromosome arms, and in its absence, cohesin complexes bind more loosely to chromatin (Inoue *et al*. 2007). In addition, it has been shown in both yeast and mammalian cells that/Chl1/DDX11 binds to the replisome (in yeast via interaction with Ctf4 and in mammalian cells through interaction with Timeless) and that this interaction is required for effective sister chromatid cohesion (Samora *et al*. 2016; Cortone *et al*. 2018). In humans, bi-allelic mutations in *DDX11* cause the rare cohesinopathy-related disease, Warsaw breakage syndrome (WABS; van der Lelij *et al*. 2010). At the cellular level, fibroblasts and lymphoblasts cultured from WABS patients display increased spontaneous and drug-induced chromosomal breakage, sister chromatid defects, and sensitivity to drugs that impede replication [DNA cross-linking agent mitomycin C and topoisomerase I inhibitor camptothecin (CPT)] but not to drugs that elicit nucleotide excision repair or cause single- or double-stranded DNA breaks (such as X-ray and UV irradiation), supporting that DDX11 plays a role in sister chromatid cohesion and replication fork stability or recovery, but not necessarily in repair of single-stranded or double-stranded DNA breaks (van der Lelij *et al*. 2010; Capo-Chichi *et al*. 2013).

In addition to identifying SL interactions, studying genetic interactions (GIs) can provide functional information on a protein's role and pathways (Kim *et al*. 2019). DDX11 plays an important role in DNA replication, repair, and sister chromatid cohesion, and the yeast ortholog, Chl1, is a highly connected SL hub that interacts with many genes involved in cancer-relevant processes (Costanzo *et al*. 2016). However, the mammalian GIs of *DDX11* have not been widely studied, prompting our analysis of the *DDX11* SL interactome in this study. In this study, we conduct an unbiased genome-wide CRISPR/Cas9 knockout (KO) screen in isogenic DDX11*-*deficient human cells. We identified many genes involved in DNA replication, repair, and sister chromatid cohesion, supporting the key role that DDX11 plays in linking DNA repair with establishment of sister chromatid cohesion and maintenance of genome stability. Our work supports another recent study using CRISPR screens in *DDX11* and *ESCO2* mutant cell lines that also shows a strong dependency of DDX11-deficient cells on cohesion regulators (van Schie *et al*. 2023).

## Materials and methods

### Cell lines

HAP1 cells are a near-haploid line derived from KBM-7 and have been previously described (Carette *et al*. 2011). HAP1 cells and HAP1 DDX11 KO cells were cultured in Iscove's Modified Dulbecco's MFBS’edium + 10% fetal bovine serum (FBS) (Invitrogen) and incubated at 37°C and 5% CO_2_.

### Western blotting

Samples for western blot were lysed in Lysis buffer (50 mM Tris-HCl, pH 7.5, 150 mM NaCl, 10% glycerol, 1% Triton X-100, and protease inhibitors), sonicated, and debris spun down at ∼18,000 × *g* at 4°C for 15 min. Samples were normalized by protein concentration using the bicinchoninic acid assay (BCA) (Thermo Fisher Scientific), run on 8% SDS-PAGE gels, and transferred to PVDF membrane (Immobilon-FL, Millipore). After probing with primary and secondary antibodies, blots were then subjected to ECL (Clarity or Clarity Max Western ECL substrate, BioRad) and visualized using a BioRad ChemiDoc MP Imager in the appropriate channel. Antibodies used for western blot were as follows: DDX11 (1:1,000; Abnova, H00001663-B01P) and α-tubulin (1:20,000: Abcam, ab18251). Secondary antibodies were either goat antimouse conjugated to HRP or goat antirabbit conjugated to HRP or Cy3 (Jackson Laboratories).

### Plasmids, primers, and single-guide RNA

For generation of KO lines, single-guide RNAs (sgRNAs; Table 1) were cloned into pSpCas9-T2A-blast, which was derived from pSpCas9-T2A-puro (Addgene # 62988). Blasticidin resistance gene was amplified from lenti-dCas9-VP64-blast (Addgene #61425) using primers OPH8968 and OPH8969 and cloned into the pCR-Blunt vector using Zero Blunt PCR Cloning Kit (Invitrogen) according to manufacturer's instructions. Site-directed mutagenesis to remove the BbsI site was performed using QuikChange Site-Directed Mutagenesis Kit (Agilent) and primers OPH9364 and OPH9365 and verified by Sanger sequencing. The modified blasticidin resistance gene was then cloned into pSpCas9-T2A-puro using EcoRI (replacing the puro gene) to obtain BPH1324. Finally, guide RNAs (gRNAs) were cloned into pSpCas9-T2A-blast using BbsI.

**Table 1.** sgRNA, primers, and plasmids used in this study.

**Identifier**	**crRNA**
STAG2	/AltR1/rArUrU rUrCrG rArCrA rUrArC rArArG rCrArC rCrCrG rUrUrU rUrArG rArGrC rUrArU rGrCrU/AltR2/
HASPIN	/AltR1/rArCrC rGrUrG rArCrC rCrCrA rArGrA rCrGrC rCrUrG rUrUrU rUrArG rArGrC rUrArU rGrCrU/AltR2/
PAXIP1	/AlTR1/rGrArGrGrUrCrArArGrUrArUrUrArCrGrCrGrGrUrGrUrUrUrUrArGrArGrCrUrArUrGrCrU/AlTR2/
**Identifier**	**Primers**
OPH9318	AATGAGATGGGTGTGAAGAGCAGG
OPH9319	TCCCAATGCACAAAGCCGAG
OPH9320	AATGAGATGGGTGTGAAGAGCAGGG
OPH9321	GGAGACCAGCCGAACATCCT
OPH9453	ATTGTTCTGGGGCGATTCCG
OPH9454	GCACATAGCCAGTGAGGGTC
OPH8968	CTGGACATGCTGATTAACGAATTCGGCAGTGGAGAGGGCAGAG
OPH8969	CGATAAGCTTGATATCGAATTCTTAGCCCTCCCACACATAAC
OPH9364	GCTGGCGACGCTGTAATCCTCAGAGATGGGGATG
OPH9365	CATCCCCATCTCTGAGGATTACAGCGTCGCCAGC
**Identifier**	**Plasmids**
BPH1324	pSpCas9(BB)-2A-Puro (PX459) V2.0
BLA371	spCas9-T2A-Blast-DDX11 Int. 5/6.2-3
BLA332	spCas9-2A-GFP-DDX11 Intron 6/7.1-1
BLA392	spCas9-T2A-BLAST-DDX11 gRNA Exon 4-1

### Generation of clonal KO lines

HAP1 parent cells were transfected with BLA371 + BLA332 (pSpCas9-T2A-Blast-DDX11 Int. 5/6.2 + pSpCas9-2A-GFP-DDX11 Intron 6/7.1) or BLA392 (pSpCas9-T2A-Blast-DDX11 gRNA exon 4) plasmids using XtremeGene 9 (Roche) according to the manufacturer's instructions. The following day, transfected cells were selected using blasticidin (Sigma) for ∼3 days, followed by replating at single-cell density in 10-cm plates. Ten to 14 days after plating, colonies were picked using cloning cylinders and transferred to a 96-well dish. Clones were passaged every 2–3 days until they reached 10 cm density, and DDX11 protein KO was tested by western blot. Parent lines and DDX11 KO clones were checked for mycoplasma before being used. Clones were also stained with propidium iodide (PI) and compared with parent cells by fluorescence-activated cell sorting (FACS) to determine ploidy.

To sequence the clones, due to the high identity between *DDX11* and other regions (*DDX12P* and *LOC642846*), genomic DNA was extracted using QuickExtract according to the manufacturer's instructions, and the relevant region was PCR amplified using primers OPH9318 + 9319 or OPH9320 + 9321 for HAP1 clones #1.1.5 and #2.1.5 and OPH9453 + 9454 for HAP1 clone #3.4.9. The PCR product was cloned into PCR_Blunt and transformed into DH5α cells, and ∼10 colonies were sequenced for each clone using M13F and M13R primers.

### Drug sensitivity assays

Cells were plated in 100-µL media in 96-well plates (6 wells per concentration). The next day, 100-µL media containing CPT, olaparib, or hydroxyurea (HU, at 2× final concentration) was added. Cells were incubated for further 3–4 days before being fixed in 3.7% paraformaldehyde and stained with Hoechst 33342, and nuclei were counted on a Cellomics Arrayscan VTI.

### CRISPR-Cas9 KO screen

CRISPR-Cas9 screen was performed as previously described (Aregger *et al*. 2020). Briefly, cells were infected with lentiviral TKOv3 library (a sequence-optimized sgRNA library of 71,090 sgRNAs targeting 18,053 human protein-coding genes with 4 sgRNAs per gene) at an MOI of ∼0.3 such that each sgRNA was represented in about 200–300 cells and then selected the following day with puromycin (2 µg/mL) for 48 h. Following selection, T0 samples were collected for determination of library representation at day 0, and the remaining cells were replated in 3 replicates maintaining >200-fold coverage of the library. Replicates were passaged every 3–4 days maintaining coverage of the sgRNA library and with 3 samples collected at T0 and all subsequent passages, until the infected population reached 16 doublings (T18). Genomic DNA was purified from T0 and endpoint samples using Promega Wizard Genomic DNA Purification kit according to the manufacturer's instructions. For each sample, sgRNA inserts were amplified from ∼50 µg of genomic DNA by a 2-step PCR reaction using primers harboring Illumina TruSeq adaptors with i5 and i7 barcodes. The sequencing libraries were gel purified and sequenced on an Illumina HiSeq 2500. Log2 fold changes (LFCs) and quantitative GI (qGI) scores were processed and calculated as in Aregger *et al*. (2020).

### Gene ontology term enrichment analysis

Enrichment of DDX11 GIs (|qGI| ≥ 0.6, false discovery rate (FDR) ≤ 5%) was analyzed using the PANTHER Overrepresentation Test (https://geneontology.org/) for either negative or positive interactions. The top 10 terms are shown for each of the hit categories.

### STAG2, HASPIN, and PAXIP1 knockdown and viability assays

The Alt-R CRISPR-Cas9 (from-integrated DNA technology [IDT]) system was used to generate STAG2, HASPIN, and PAXIP1 knockdown in HAP1 and *DDX11* KO cell lines. Briefly, crRNAs targeting *STAG2, HASPIN*, or *PAXIP1* (see Table 1 for sequences) were mixed in equimolar ratios with tracrRNA (IDT) to form the sgRNA complex. A total of 1 μM sgRNA was complexed with 1 μM purified Cas9 (IDT), combined with RNAiMAX transfection reagent (Thermo Fisher Scientific) and mixed with cells.

HAP1 and *DDX11* KO cells were seeded at 80,000 cells/well in a 24-well plate, reverse transfected as described above, and incubated for 48 h. Adherent cells were stained with crystal violet and resuspended in 10% acetic acid in methanol, and the OD570 was measured. HAP1 and *DDX11* KO cells were reverse transfected as described above and seeded at 40,000 cells/well in an opaque 96-well plate and incubated for 48 h. A total of 150 μL of CellTiter-Glo reagent (Promega) were added to each well, and fluorescence intensity was measured at 520 nm.

HAP1 and DDX11 KO cells were seeded at 10,000 cells/well in a 24-well plate, incubated for 24 h, treated with CHR-6494 trifluoroacetate, and incubated for an additional 72 h. Adherent cells were stained with crystal violet and resuspended in 10% acetic acid in methanol, and the OD570 was measured.

## Results

### Generating DDX11 KO HAP1 clones

**Fig. 1.**
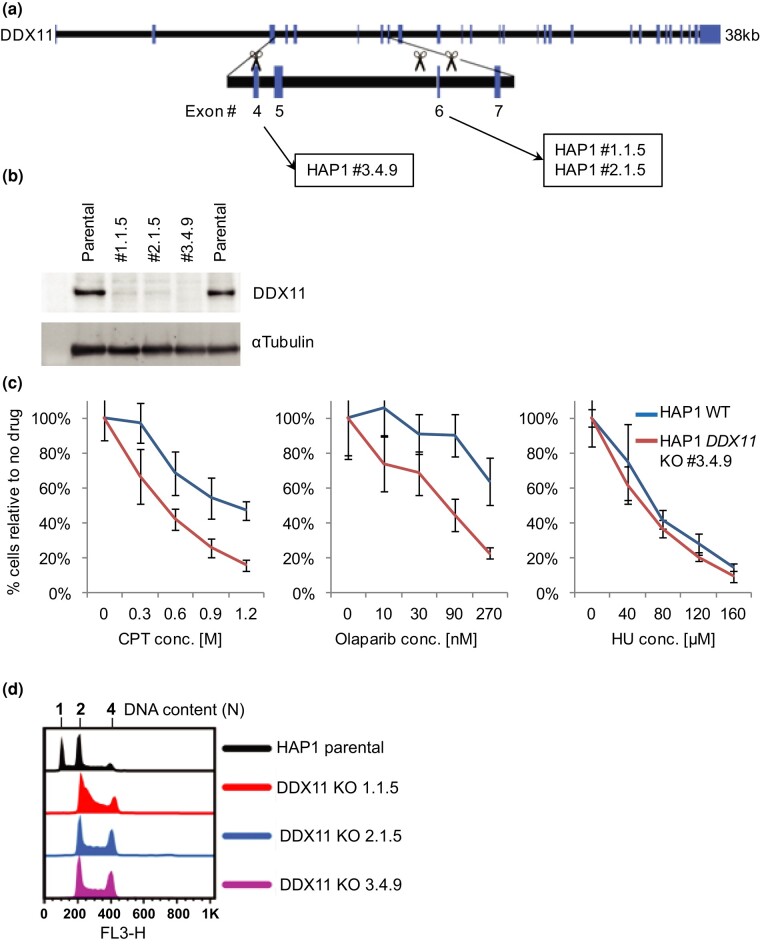
Engineering *DDX11* CRISPR/Cas9 KO lines. a) DDX11 genomic structure and strategy for making KO lines. Rectangular boxes represent exons, and the connecting lines represent intronic DNA. Scissors depict cleavage locations of sgRNAs selected, and boxes indicate which clones were derived from each strategy. b) Western blot analysis of DDX11 in promising HAP1 *DDX11* KO clones. Alpha-tubulin is shown below as a loading control. c) HAP1 parental and lead candidate *DDX11* KO cell lines treated with the indicated doses of CPT, olaparib (Ola), HU, or DMSO in 96-well format. After 3 days, cell numbers were quantified by nuclei counting using Cellomics Arrayscan VTI. Data are presented as mean ± SD from 6 replicates. d) FACS analysis of PI-stained DNA content in the indicated cell lines to determine ploidy.

To generate *DDX11* KO cell lines, we chose the human near-haploid cell line HAP1 as a model system, given the relative ease of generating KO mutations in this background (Carette *et al*. 2011). Targeting *DDX11* is challenging as it is located in a complex repetitive region on chromosome 12. Several highly related *DDX11* pseudogenes, including *DDX12P* and *LOC642846*, exist on chromosome 12, and members of the *DDX11L* family map to multiple chromosomes (Amann *et al*. 1996; Costa *et al*. 2009). While we explored various strategies involving 1 or 2 sgRNAs (Fig. 1a), screening 47 clones for DDX11 protein KO, ultimately a single sgRNA strategy, produced clone 3.4.9. Clone 3.4.9 appeared to contain 2 editing events when aligned to *DDX11* sequence (an insertion of a single C or insertion of CT—both of which create a frameshift and early termination). However, when aligning the sequences to *DDX12P* and *LOC642846* pseudogenes as well as to *DDX11*, the single C insertion is most likely at the *DDX11* locus, and the CT insertion is more likely to be at *DDX12P* or *LOC642846* loci. Therefore, it seems that this clone was also derived from a haploid clone that diploidized after the genome editing event. This DDX11 KO in clone 3.4.9 appeared to be complete by western blot (Fig. 1b) and exhibited sensitivity to CPT and olaparib but not HU (Fig. 1c). This is consistent with published data that human lymphoblastoid cells lacking DDX11 are sensitive to CPT (a topoisomerase I inhibitor) and to PARP inhibitors, but DDX11/Chl1 is largely dispensable for cell survival in chicken DT-40 and budding yeast cells following exposure to HU (a ribonucleotide reductase inhibitor; van der Lelij *et al*. 2010; Laha *et al*. 2011; Stoepker *et al*. 2015; Abe *et al*. 2018). HAP1 cells often contain a subpopulation of cells that spontaneously switch to a diploid state during normal culturing and often become fully diploid within 10–20 passages after CRISPR/Cas9 editing (Beigl *et al*. 2020). To test the ploidy of the *DDX11* mutant clones, cells were stained with PI and compared with parental cells by FACS analysis. Several candidate KO clones, including 3.4.9, had become diploid compared with the parental line (Fig. 1d). In summary, clone #3.4.9 demonstrated the cleanest KO by western blot and the strongest expected DDX11 KO drug sensitivity and was selected for the CRISPR/Cas9 KO screen.

### Genome-wide CRISPR/Cas9 KO screen of DDX11-deficient cell lines

**Fig. 2.**
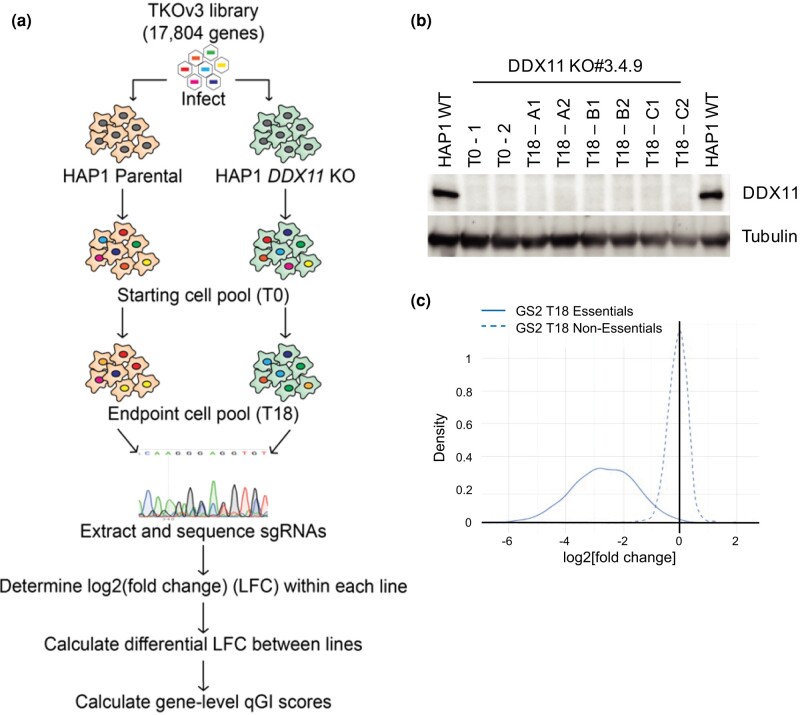
CRISPR/Cas9 screen for the identification of GIs in *DDX11* KO HAP1 cells. a) A schematic of the screen. DDX11 KO and WT parental cells were infected with a lentiviral genome-wide CRISPR gene KO library (TKOv3), and gRNA abundance was determined. LFC was calculated for each gRNA within each cell line, and then the differential LFC between WT and KO cells was calculated. Finally, a series of normalization steps and statistical tests were applied to these data to generate gene-level qGI scores and FDRs. b) Western blot of DDX11 from all cell lysates collected during and after the screen to confirm stable DDX11 KO. Tubulin is shown as a loading control. c) Screen quality control statistics showing differences in gRNA representation for core essential (solid) and nonessential (dotted) controls genes targeted within the screen. Nonessential genes center around 0, while core essential genes appear strongly negative.

We conducted a genome-wide CRISPR/Cas9 screen using the TKOv3 gRNA library, which contains ∼71,090 gRNAs that target ∼18,000 human protein-coding genes, most of them targeted by 4 unique gRNAs (Hart *et al*. 2017; Aregger *et al*. 2019). The relative abundance of individual gRNAs was compared between the screen start (T0, following infection and selection) and end (T18, after 16 doublings) providing an estimate of single-mutant fitness, whereas the relative abundance in *DDX11*-mutated cells provides an estimate of double-mutant fitness (schematized in Fig. 2a). The GIs were scored using a qGI score that measures the strength and significance of the interaction by comparing the relative abundance of gRNA in the mutant cell line with the relative abundance of the same gRNA in an extensive panel of 21 wild-type HAP1 screens, after the removal of frequent flyers and batch correction (Aregger *et al*. 2019). Negative interactions reflect genes whose gRNAs are significantly decreased in the *DDX11*-mutated line relative to the control wild-type panel, whereas positive interactions reflect genes with increased gRNA abundance in *DDX11*-mutated line compared with the control wild-type panel.

**Fig. 3.**
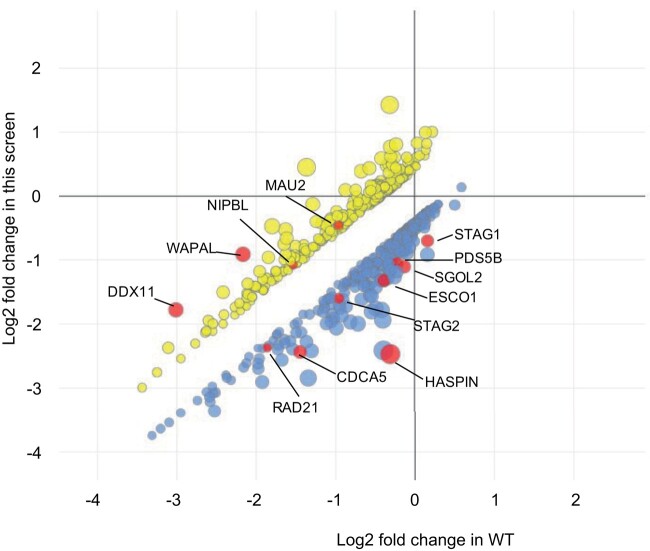
*DDX11* negative and positive GIs. A scatterplot illustrating the fitness effect (LFC) of 642 genes in *DDX11* KO versus WT parental HAP1 cell line, which exhibited a significant GI (|qGI| > 0.4, FDR < 0.2). A total of 324 negative (blue, lower cluster) and 320 positive (yellow, upper cluster) DDX11 GIs are shown. Node size corresponds to a combined score reflecting both the qGI and the FDR. Selected genes belonging to the cohesin complex or affecting sister chromatid cohesion are highlighted in red (and labelled with lines and gene names).

DDX11 KO was maintained throughout the screen, and there was no reversion of the mutation to restore DDX11 levels (Fig. 2b). To evaluate screen performance, LFC of essential genes and nonessential genes were analyzed and compared with a reference set of core essential and nonessential genes as previously described (Hart *et al*. 2017). The screen robustly distinguished the reference set of essential genes from nonessential genes, indicating a high-quality screen (Fig. 2c). Analysis of the *DDX11* mutant-specific hits identified 324 negative GIs (NGIs) at a cutoff of qGI < −0.4 at FDR ≤ 0.2 and 320 positive GIs (PGIs) at a cutoff of qGI > 0.4 at FDR ≤ 0.2 for DDX11-KO cells. As expected, multiple genes associated with the cohesin complex and sister chromatid cohesion were identified as both positive and negative genetic interactors of DDX11-KO (Fig. 3). One of the strongest positive interactions was *DDX11* itself, which supports the quality of the screen; gRNAs in the library targeting *DDX11* cause impaired growth in the wild-type cells, but not the DDX11 KO cells as the protein is not expressed, and this manifests in the screen results as a positive interaction.

### Pathway analysis confirms cohesion-associated DDX11 dependencies

**Fig. 4.**
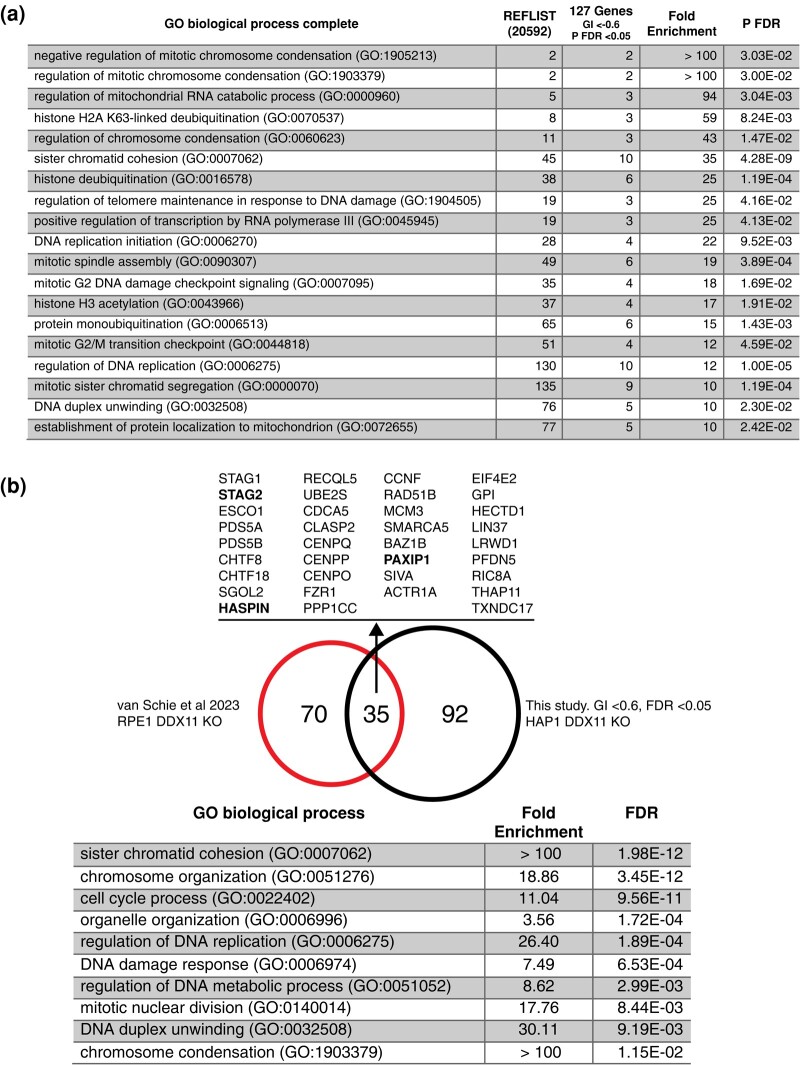
The GO enrichment of dependencies and comparison of DDX11 GIs in HAP1 and RPE1 cell lines. a) A summary of top enriched biological processes (qGI| > 0.6, FDR < 0.05) determined using the Panther DB (https://geneontology.org/). b) A comparative analysis of *DDX11* GIs in HAP1 and RPE1 cell lines. GIs derived in the RPE1 cell line from van Schie *et al*. (2023) (smaller red circle). GIs derived in the HAP1 cell line in this study (larger black circle). Thirty-five negative GIs were shared between the experiments in HAP1 and RPE1. These shared interactions were enriched for cohesin, cell cycle, and DNA replication factors based on biological processes using the Panther DB (https://geneontology.org/).

To provide further insight into the functional categories of genes identified, we performed gene ontology (GO) term enrichment analysis using PantherDB PANTHER Overrepresentation Test (Mi *et al*. 2021). We first looked at the negative GIs, which reflect genes that are SL or synthetic sick with DDX11 KO. For this analysis, we set a more stringent cutoff of qGI > 0.6 to compute enrichments of the strongest hits. The top enriched terms for biological process GO terms are shown in Fig. 4a. Consistent with the known role of the DDX11 helicase, the enriched terms were associated with the cell cycle, DNA repair, and chromosome cohesion and segregation. Interestingly, among the top enriched terms that were associated with DNA damage response and cohesion related, supporting the hypothesis that DDX11 inhibition may be a good therapeutic target in cancer cells, many of which carry defects in DNA repair pathways.

A recent paper reported negative GIs for a DDX11 KO in immortalized RPE1 cell lines (van Schie *et al*. 2023). GIs are often context dependent with relatively few interactions conserved between different cell lines with the same genetic KO query gene. For example, a comparison of GIs with STAG2 KO in 3 different cell lines found only one interaction common to HAP1, RPE1, and tumor-derived H4 cell lines (Bailey *et al*. 2021). To find common GIs with loss of DDX11 and to corroborate the GIs found in the HAP1 CRISPR screen, we compared the top negative GIs reported by van Schie *et al*. (2023) with the top negative interactions found in our CRISPR screen. Of the 105 top negative interactions with DDX11 RPE1 (van Schie *et al*. 2023) and the 127 top negative interactions, we found in DDX11 KO HAP1 cells, 35 were common to both screens and were highly enriched for cohesion-associated genes (Fig. 4b).

### Validation of DDX11–cohesion GIs

**Fig. 5.**
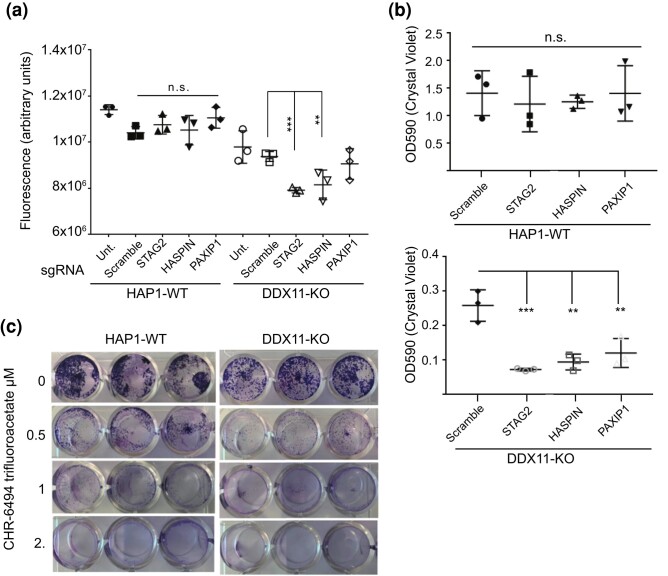
The validation of CRISPR screen hit synthetic sickness with *DDX11*-KO. a) Cell viability by the CellTiter Glo method after transfection of the indicated gRNA. Each condition was measured in triplicate, and ANOVA was used to compare all means with the scramble-treated HAP1-WT cell line. ****P < 0.0001, ***P < 0.001, *P < 0.05. b) Crystal violet staining as a measure of viability in the indicated treatment conditions. Each condition was measured in triplicate, and the results of ANOVA comparing with the respective scramble treated are shown. ****P < 0.0001, ***P < 0.001, **P < 0.01. c) Crystal violet staining for viability of technical triplicates for the indicated cell line exposed to the indicated drug concentration of CHR-6494 trifluoroacetate.

Finally, we sought to confirm that the observed interactions could be reproduced by direct tests of fitness. We chose to focus on STAG2 because of its importance as a tumor-suppressor gene lost in various cancers, PAXIP1 a recently identified cohesin regulator, and the Haspin kinase since it may be targetable with small molecules (Liang *et al*. 2018; Bailey *et al*. 2021; Mayayo-Peralta *et al*. 2023). We used CRISPR sgRNAs targeting *STAG2, GSG2* (the catalytic subunit of Haspin), or *PAXIP1* to generate knockdowns. Cellular fitness in gRNA transfected cells was measured in comparison with untreated or scrambled sgRNA controls using 2 independent assays. First, we quantified metabolically active cells using the CellTiter Glo cell viability assay and found that *STAG2* and *HASPIN* sgRNA treatment significantly reduced cell viability in the *DDX11*-KO relative to a scrambled control but did not reduce fitness in the parental cell line (Fig. 5a). We also used crystal violet staining and colorimetric quantification as a proxy for cell viability. *DDX11* KO cells were less viable/adherent compared with the parental lines (note the different *Y*-axis scales); however, treatment with the *STAG2, HASPIN*, or *PAXIP1* sgRNA significantly reduced crystal violet staining in the KO but not in the wildtype (WT) cell lines (Fig. 5b). Finally, the HASPIN kinase inhibitor, CHR-6494 trifluoroacetate was tested and also qualitatively reduced the *DDX11*-KO cell growth compared with WT (Fig. 5c). Together, these experiments validate several cohesin-related hits from our primary screen, confirming that *DDX11*-KO cells are highly dependent on intact sister chromatid cohesion to survive.

## Discussion

Studying GIs expands our understanding of their molecular role(s) and therapeutic potential. The DDX11 helicase plays an important role in DNA replication, repair, and sister chromatid cohesion. The yeast *DDX11* homolog, *CHL1*, is a highly connected SL hub with many genes involved in cancer-relevant processes, but the mammalian GIs of *DDX11* are only recently being defined (Faramarz *et al*. 2020; van Schie *et al*. 2023). The goal of this study was to conduct an unbiased screen in isogenic DDX11 KO cells to provide additional functional and therapeutic information.

For our screen, we chose to use HAP1 lines with/without DDX11. In a previous study, *DDX11* was defined as an essential gene in HAP1 cells using a gene-trap method to systematically inactivate genes (Blomen *et al*. 2015). This essentiality is supported by data from the DepMap project (a large-scale project aiming to systematically identify genetic and pharmacologic dependencies in a large panel of cancer lines), in which *DDX11* is defined as a “common essential” gene (Pacini *et al*. 2021). Given the presence of highly similar *DDX11* pseudogenes, it is possible that the “essentiality” of *DDX11* in pooled CRISPR screens is, in part, a byproduct of multiple CRISPR-induced double-stranded breaks in the genome that reduce viability and are selected against in pooled competitive growth conditions. In the case of our generated clones, cells were edited and plated at single-cell density until the formation of a colony. Under these conditions, even cells with fitness defects may be able to survive and form colonies. Other groups have also managed to KO DDX11 in human cells in HeLa, U2OS, and RPE1 cells using CRISPR/Cas9 genome editing (Jegadesan and Branzei 2021; van Schie *et al*. 2023).

Our screen identified multiple GIs (both positive and negative) with genes involved in sister chromatid cohesion or cohesion establishment and maintenance (Fig. 3). Our genetic screen was in a single-cell type, HAP1, and many GIs are cell line specific and are not broadly shared across cell lines. To identify common GIs with DDX11 KO, we compared our strong negative interactions with those from a CRISPR screen in immortalized retinal pigment epithelium (RPE) cells (van Schie *et al*. 2023). There was significant overlap (35/105 negative interactions) between the strong negative interactions, and the common interactions were enriched in genes involved in chromatin cohesion. This comparison reinforces the findings in both datasets.

The GIs observed reflect the underlying molecular roles of DDX11. For example, sororin (CDCA5), WAPAL, and PDS5 form a cohesin-regulator complex in vertebrates, in which sororin and WAPAL antagonize each other by competing for binding to a specific site on PDS5 to regulate association of cohesin with chromatin. This complex positively or negatively regulates the association of cohesin with chromosomes, depending on which protein binds to PDS5. PDS5–sororin complex maintains sister chromatid cohesion, whereas PDS5–WAPAL dislodges cohesin from chromatin (Zhang *et al*. 2021). In our screen, both *CDCA5* and *PDS5B* were identified as negative GIs, whereas *WAPAL* was identified as a positive GI. This is consistent with the known role of DDX11 in establishing and maintaining sister chromatid cohesion. In the absence of DDX11, cohesion is less robust, and further dissociation through the loss of PDS5 or sororin may be detrimental to the KO cells. On the other hand, in wild-type cells, the loss of WAPAL is detrimental as it leads to increased cohesin on the DNA, whereas in the DDX11 KO cells, this effect is counteracted by the loss of cohesion due to the loss of DDX11 activity. Another protein that ties into the regulation of cohesin maintenance versus removal is the kinase HASPIN (GSG2). *HASPIN* was one of the strongest negative GIs identified in the screen, and we confirmed the interaction with potent HASPIN kinase inhibitors. HASPIN binds and phosphorylates WAPAL, directly inhibiting the interaction of WAPAL with PDS5B. Cells expressing a WAPAL-binding-deficient mutant of HASPIN or treated with HASPIN inhibitors show centromeric cohesion defects (Liang *et al*. 2018). In contrast, HASPIN also binds to PDS5B, and KO of HASPIN or disruption of HASPIN-PDS5B interaction causes weakened centromeric cohesion and premature chromatid separation, which can be reverted by centromeric targeting of a short fragment of HASPIN containing the PDS5B-binding motif or by prevention of WAPAL-dependent cohesin removal (Zhou *et al*. 2017). Together, the interactions identified support a central role for DDX11 in regulation of cohesin establishment/protection versus removal.

One of the goals of this screen was to identify potential genetic features of tumors that might be treated with DDX11 inhibition. The pattern of interactions identified suggests DDX11 inhibition may be therapeutic for tumors exhibiting a cohesin-dysregulation/premature separation phenotype. This builds upon the concept of expanding the definition of clinically relevant SL from a gene/gene (or inhibitor) negative interaction to a phenotype or pathway + inhibitor interaction, similar to the recent evidence for expansion of PARP inhibitors from treatment of tumors carrying *BRCA1/2* mutations to tumors displaying a “BRCAness” phenotype (Lord and Ashworth 2016).

In addition to potential therapeutic potential, studying GIs can provide information on the biological role of DDX11. This screen identified multiple genes important for sister chromatid cohesion, as well as genes involved in DNA repair, providing further support for the conservation of DDX11's role from yeast to human, and strengthening the idea of DDX11 inhibition as a therapeutic for cancer with cohesion defects. Of course, such cancers would need to be identified by the presence of a biomarker (similar to *BRCA1/2* mutations as an indication for treatment with PARP inhibitors). Even in the absence of a defined genotypic vulnerability, such tumors could potentially be identified by a phenotypic assay of cohesion defects.

## Supplementary Material

jkae052_Supplementary_Data

## Data Availability

All data necessary to reach our conclusions are included within the manuscript and Supplementary material. Unique reagents, cell lines, and other resources are available through contacting the corresponding authors. Supplemental material available at G3 online.
