# Supplementary material for: Mapping of DDX11 genetic interactions defines sister chromatid cohesion as the major dependency
Source: G3 (Bethesda). 2024 Mar 13;14(5):jkae052. doi: 10.1093/g3journal/jkae052 (PMC11075568; doi:10.1093/g3journal/jkae052)
Supplement: jkae052_Supplementary_Data [file jkae052_supplementary_data.zip › Table_S1_Legend_G3-2023-404784.docx]

**Supplementary Information**

**Table S1. Complete list of gene level scores arising from DDX11-KO CRISPR screen.**
